# Mass Spectrometry in Pharmacokinetic Studies of a Synthetic Compound for Spinal Cord Injury Treatment

**DOI:** 10.1155/2015/169234

**Published:** 2015-05-19

**Authors:** María Sánchez-Sierra, Isabel García-Álvarez, Alfonso Fernández-Mayoralas, Sandra Moreno-Lillo, Gemma Barroso García, Verónica Moral Dardé, Ernesto Doncel-Pérez

**Affiliations:** ^1^Grupo de Química Neuro-Regenerativa, Hospital Nacional de Parapléjicos, Servicio de Salud de Castilla La-Mancha (SESCAM), Finca La Peraleda s/n, 45071 Toledo, Spain; ^2^Instituto de Química Orgánica General, CSIC, Juan de la Cierva 3, 28006 Madrid, Spain; ^3^Servicio de Proteómica, Hospital Nacional de Parapléjicos, Servicio de Salud de Castilla La-Mancha (SESCAM), Finca La Peraleda s/n, 45071 Toledo, Spain

## Abstract

The studies of drugs that could constitute a palliative to spinal cord injury (SCI) are a continuous and increasing demand in biomedicine field from developed societies. Recently we described the chemical synthesis and antiglioma activity of synthetic glycosides. A synthetic sulfated glycolipid (here IG20) has shown chemical stability, solubility in polar solvents, and high inhibitory capacity over glioma growth. We have used mass spectrometry (MS) to monitor IG20 (*m*/*z* = 550.3) in cells and tissues of the central nervous system (CNS) that are involved in SCI recovery. IG20 was detected by MS in serum and homogenates from CNS tissue of rats, though in the latter a previous deproteinization step was required. The pharmacokinetic parameters of serum clearance at 24 h and half-life at 4 h were determined for synthetic glycoside in the adult rat using MS. A local administration of the drug near of spinal lesion site is proposed.

## 1. Introduction 

Trauma injuries in the central nervous system (CNS) have social and economic relevance worldwide. In this sense the spinal cord injury (SCI) have an incidence between 25 and 30 new cases per million population per year in Spain, which represents more than a thousand new cases annually. The Paraplegic's Hospital of Toledo has observed that 80% of acute patients correspond to young individuals, between 15 and 39 years of age. This hospital reported that traumatic accidents were the leading cause of hospitalization in 2013 in relation to no traumatic SCI diseases (tumors, spinal inflammation, demyelinating diseases, etc.) [1]. The finding of palliatives or a cure for SCI constitutes a continuous challenge in neuroscience. Nowadays a more effective treatment is a crucial necessity for SCI patients with a higher lifespan than in the last century.

The compounds that mimic neurotrophin signaling and overcome the pharmacokinetic and side-effect barriers may have greater therapeutic potential for SCI treatment. Particular emphasis is placed on small molecules that are able to modulate neurotrophin function in this pathology of the CNS. These alternative strategies show promise in preclinical studies, with some advances into clinical development [2].

Pharmacokinetics studies the effects of biological systems in a determined drug. The absorption, distribution, metabolism, and excretion of drugs from the biological systems are the principal objectives of this discipline of pharmacology. The SCI is associated with many physiological changes that can affect disposition of drugs. In general, volume of distribution is significantly higher in the SCI population compared with the non-SCI population; however, clearance and half-life may be larger or no different. In some cases the intramuscularly administration of a drug results in absence of difference in bioavailability when the dose is administered below the level of the injury; but absorption appears to be slower in patients with SCI compared with the non-SCI population [3]. The influence of the pathophysiology of spinal cord injury on gastrointestinal motility appears to be reflected in an impairment in the bioavailability of drugs which are passively absorbed and which require an intact postprandial gastric emptying to ensure efficient absorption [4]. In preclinical studies for treatment of SCI the presence in blood circulation of intact compound can be guaranteed by intravenous administration way [5].

Here we use mass spectrometry (MS) by matrix-assisted laser desorption/ionization (MALDI) to monitoring the synthetic compound IG20 in its sulfuric form (Figure 6) in the CNS tissues. We observed that microglia and astroglial cells, involved in SCI lesions, are inhibited by this compound. The present studies use MS to detect the glycoside in serum and CNS tissue homogenates of rats. Additionally, we followed the locomotion recovery after IG20 injection in rats with moderate contusion in spinal cord as part of preclinical studies. The IG20 compound is currently used in experimental investigations at HNP aimed at developing a new therapy for SCI.

## 2. Materials and Methods

### 2.1. Animals

All experiments followed European Council directive number 86/609/CEE and the U.S. Department of Health guidelines to limit pain and discomfort to experimental animals. This study was approved by local ethical committee for animal welfare. Wistar rats, bred and maintained at the house animal of the Paraplegics Hospital, were used in this study.

### 2.2. Cell Cultures and Compounds

Primary glia cells were derived using standard procedures. These cells were prepared from cerebral cortices of new born Wistar rat pups. After incubation for 9–11 days, the astrocytes were purified by 12 h shaking to separate microglia and oligodendrocytes progenitor cells. The murine microglia cell line BV-2 was purchased to Banca Biologica e Cell Factory (IST, Genova) and N13 cell line was gently donated by Dr. ML de Ceballos (Cajal, Madrid). The compound IG20 (C_26_H_48_KNO_9_S) was synthesized in the Organic Chemistry Institute, Madrid, and conserved in lyophilized form until use. The biological assays with these compounds were performed in Paraplegics Hospital, Toledo.

### 2.3. Cell Inhibition Assay

Isolated cells were seeded in DMEM plus 10% FCS at 1 × 10^4^ cells/well and allowed to attach for 6 h. The medium was changed to serum-free DMEM and the cells were incubated for 36 h, afterwards, the medium was replaced by DMEM plus 1% FCS, containing the inhibitor compound and incubated for 48 h. Then cell proliferation was measured by incubation (4 h) of cells with MTT (3-(4,5-dimethylthiazol-2-yl)-2,5-diphenyltetrazolium bromide) to form insoluble formazan. The formazan crystals were solubilized by lysing solution (0.1% SDS in 0.01 M HCl) and the plate incubated 18 h at 37°C. The optical density value at 570 nm was recorded by plate reader (Ultra 384, TECAN). Inhibition was calculated using the following formula:

(1) $$\begin{array}{c} \left(\%\;\text{inhibition}\right)=100-100\left[\frac{\left(X-B\right)}{\left(A-B\right)}\right], \end{array}$$

where *A* were absorbance value, corresponding to cells maintained in DMEM plus 10% FCS (high mitosis control); *B* were the absorbance value in cells in serum-free medium (low mitosis control), and *X* corresponded to absorbance value in cells treated with inhibitor. Dose-response plots of percent inhibition versus concentration were obtained from triplicate samples and adjusted to sigmoidal curves, from which values of the 50% inhibitory concentration (IC_50_) were calculated [6].

### 2.4. SCI Surgery and Drug Administration to Animals

Female Wistar rats (~200 g) were deeply anesthetized with pentobarbital (45 mg/kg) and xylazine (10 mg/kg). Once the animal was areflexic, the lower thoracic spinal cord was exposed by performing a laminectomy at the T9 vertebral level. The animal was then positioned and secured into the frame of the IH Impactor by clamping the T8 and T11 spinous processes and the rats received a moderate (200 kD) contusion with the Infinite Horizon (IH) device at T9. The anesthetized animals (*n* = 5) received intravenous injections of IG20 (30 mg/kg) or saline (*n* = 5) at same day of laminectomy, at 2nd and 6th day. After compound administration, the operated rats were placed on a heating pad maintained at 37°C. Postoperatively, rats received subcutaneous injections of analgesic (Buprenex, 0.03 mg/kg), antibiotic (Baytril, 2.5 mg/kg), and saline to hydrate. Bladders were manually pressed twice daily until recovered the reflex bladder function.

To follow IG20 clearance in serum, adult rats of two months of age (*n* = 12) were anesthetized by isoflurane (Baxter, Illinois) inhalation and a unique intracardiac injection of IG20 (30 mg/kg) was practiced. The IG20 injected animals were divided in 4 groups (3, 6, 12, and 24 h). The control group (*n* = 3) were injected with saline and processed at 24 h. At the end of experiments the assayed animals were euthanized by CO_2_ inhalation, brain and spinal cords were dissected for further analysis.

### 2.5. BBB Locomotor Rating Scale

Hindlimb motor function was assessed using the BBB locomotor rating scale [7]. Two examiners were positioned across from each other to observe both sides of the animal during the 4 min testing session. We did not test rats in the same day in order to avoid the possibility of fatigue influencing BBB scores. Spinally injured animals were then allowed to recover for 1, 3, 7, 14, 21, and 28 days (*n* = 5 per treatment group, saline or IG20, at each survival time). Animals had* ad libitum* access to both food and water.

### 2.6. Sample Processing

A volume (1 mL) of total blood sample from treated animals was allowed to stand for 30 minutes at RT to promote clot formation, which was discarded. The supernatant was centrifuged at 3500 rpm for 10 minutes. The resulting clean serum was conserved at −20°C for mass spectrometry analysis.

At same time the brain and spinal cords were dissected from IG20 injected animals immediately after euthanasia. These neural tissues were homogenized independently using Dounce device and dissolved in phosphate buffer (KH_2_PO_4_ 20 mM/PMSF 1 mM, pH = 6.8) at 1 : 1. In positive controls the homogenized tissues were incubated with IG20 (500 *μ*g) in rotation by 1 h at 4°C. The mixture was centrifuged at 16500 g, 30 min at 4°C. The pellet was resuspended in phosphate buffer for deproteinization with Proteinase K (1 mg/mL). The total glycolipids were extracted with 4 volumes of tetrahydrofurane (LAB-SCAN) and centrifuged 15 min. The supernatant was mixed 1 : 1 with isopropyl ether, (Sigma), centrifuged 15 min. The aqueous phase with total glycolipid extract was lyophilized. For desalting and concentrating purposes a LiCHrout RP-18 column (Merck) was equilibrated with CHCl_3_/MeOH/H_2_O (3 : 48 : 47). After MeOH elution the final extract of glycolipids was obtained.

### 2.7. Mass Spectrometry

The pure compounds, rat serum samples, and total glycolipids from brain or spinal extracts were dissolved in MeOH and analyzed by mass spectrometry (MS). A 10 *μ*L aliquot of serum controls was doped with 5 *μ*L of IG20 at 0.001 *μ*g/*μ*L and 0.0001 *μ*g/*μ*L concentrations. Solutions of the synthetic compound at an interval of a 0.1 ng/*μ*L–0.2 *μ*g/*μ*L were mixed in a vial with the matrix in two different ratios of sample: matrix (1 : 1 and 1 : 10). For doped control serum samples, only the 1 : 1 ratio was used. A 5 *μ*L extract of glycolipids was mixed with the same amount of matrix (2,5-dihydroxybenzoic acid (Sigma Aldrich) 10 mg/mL, EtOH 20%). 0.5 *μ*L of each mixture were deposited using the dry droplet method, onto a 384 Opti-TOF 123 × 81 mm MALDI plate (Applied Biosystems) and allowed to dry at room temperature. The way of sample acquisition in MALDI-TOF analysis is shown (see Supplementary Figure 1 in Supplementary Material available online at http://dx.doi.org/10.1155/2015/169234).

MALDI-MS data were obtained in an automated analysis loop using a 4800 Plus MALDI TOF/TOF Analyzer (Applied Biosystems). MS reflector negative ion mode with automated acquisition of 350–1500* m/z* range was used with 1000 shots per spectrum. The shooting of three random shapes on the position of the plate were accumulated (uniform, centered and bias end). The laser used is a Nd: YAG laser of 355 nm wavelength, at 200 Hz laser frequency. Spectra were adjusted with external calibration using 4700 Cal Mix, (Applied Biosystems).

### 2.8. Statistical Analysis

Each point value of proliferation inhibition curves was obtained by triplicates and the experiments were repeated three times at least. The values in table and bar graph are shown as ±Mean Square Error (MSE). For BBB scale analysis a paired *t* test was used, and *P* value <0.05 was considered as significant difference.

## 3. Results

### 3.1. The Mass Spectrometry Profile of IG20 Is Obtained in Negative-Ion Mode

**Figure 1:**
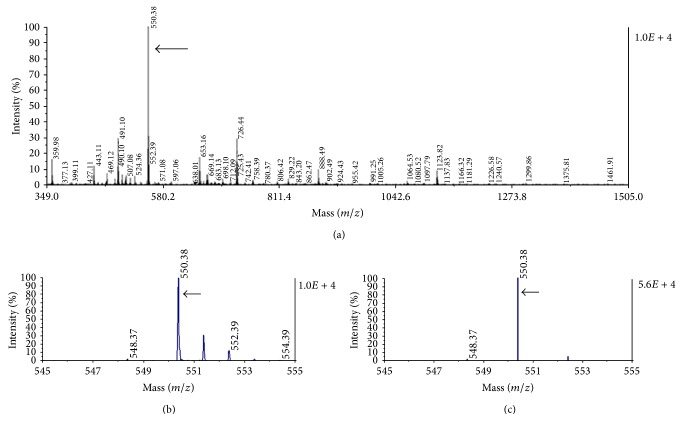
Mass spectrum for the IG20 glycoside in negative-ion mode. The MALDI mass spectrum for IG20 (a), its mass isotopic distribution (b) and deisotoping for IG20 peak (c). Arrows pointed the mass peak for IG20 at 550.3* m/z*.

Saccharides and oligonucleotides are usually analyzed under negative ionization conditions [8, 9]. We obtained the IG20 glycoside as a unique final product of a chemical synthesis process [6]. The ESI-MS (electrospray ionization-mass spectrometry) spectrum, obtained by negative-ion mode shown a high purity and 550.3* m/z* value for IG20 compound at the end of synthesis (Figure 2 of Supplementary Material). The anionic charge due to sulfate group in the sugar moiety (Figure 6) is suitable to use the negative-ion mode for IG20 (Figures 1(a)–1(c)). Using a more restricted range in the mass spectrum gave the isotopic distribution corresponding to ionized IG20 (Figure 1(b)). When automated algorithm is used the deisotoping [10] of IG20 peak is obtained, this results in a unique IG20 peak (550.3* m/z*) that can be easily detected from background, in case of more complex mixtures (Figure 1(c)).

**Figure 2:**
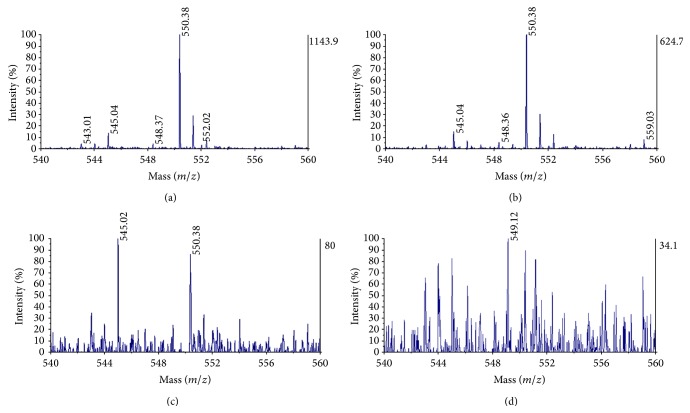
Limit of detection in solution for IG20 by MALDI-TOF MS. The mass spectrum for IG20 at 500 pg/*μ*L (a), 250 pg/*μ*L (b), and 100 pg/*μ*L (c) is shown. Below these concentrations the IG20 peak was undetectable from background (d). The sample and DHB matrix in ratio of 1 : 1; the mass peak for IG20 at 550.3* m/z*.

For selection of range of analysis the* m/z* scale was calibrated by standard sample of peptides mixture near the mass for IG20 (589, 82 for the potassium salt) then mass correction was applied. In mass spectrometry spectrum a clear and high signal for IG20 anion was observed at mass of 550.3 (Figures 2(a)–2(c)), molecules of lower mass corresponding to ionization of matrix were observed also (Figure 2(d)). In order to establish the concentration ranges that could be detected, different standard dilutions were prepared, using 2,5-dihydroxybenzoic acid (DHB) as matrix in an organic/aqueous solvent to avoid of drop migration on the surface. This leads to an excellent spectral resolution and very good signal to noise ratio in the corresponding mass spectra. These dilutions were mixed with DHB matrix in ratio 1 : 1 for sample: matrix to get the best signal quality. The lowest concentration detected for IG20 compound was about 100 pg/*μ*L (Figure 2).

### 3.2. The IG20 Glycoside Inhibited the Proliferation of Microglia and Astroglia Cells

Two cell lines of murine microglia (BV-2, N13) were used, as well as primary astrocytes isolated from cerebral cortices of P0 postnatal rats. The cells were maintained in DMEM plus 10% FBS, grown and treated for inhibition assay of cellular proliferation, as we published previously [6]. In both cell lines of microglia an effective inhibition of proliferation was observed corresponding to IC_50_ of 21.0 *μ*M and 5.7 *μ*M for N13 and BV-2 cells, respectively. In summary the inhibitory activity of IG20 is effective in both microglial cell lines and its IC_50_ being very similar to that obtained for astrocytes treated by IG20, used as positive control in these assays (Table 1).

### 3.3. The IG20 Clearance in Serum Followed by Mass Spectrometry

**Figure 3:**
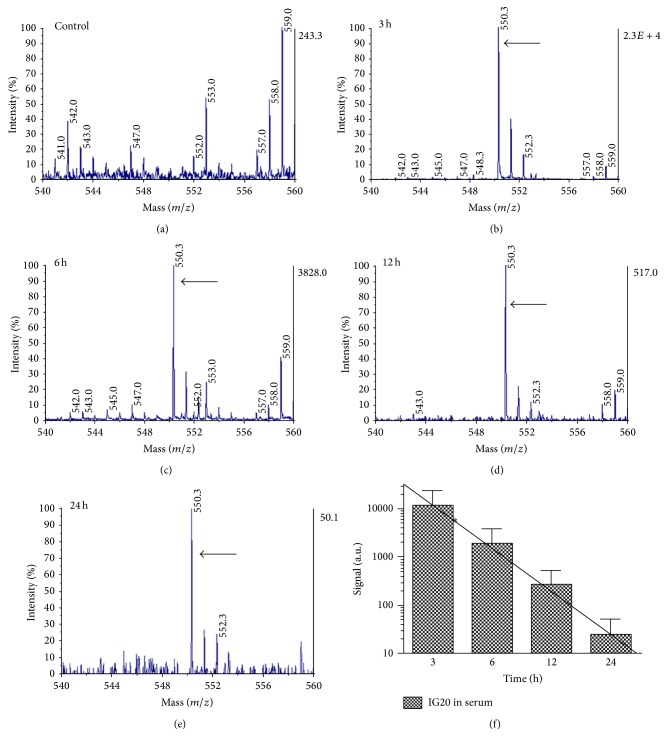
The clearance of IG20 was registered by mass spectrometry in rat serum samples after the IG20 injection. In control group (a) no signal for IG20 was observed, but in injected animals a signal of mass at 550.3 (see arrows) corresponding to IG20 glycoside was detected at 3, 6, 12 and 24 h ((b), (c), (d), and (e)). The IG20 clearance was followed by the average of signal values of IG20 peaks in each serum sample by the time shown (f). The solid line represents a linear regression in which a half-life of ~4 h (∗) of intact IG20 was inferred (f).

The mass of 550.3 for IG20 was detected in serum isolated from blood of rats after intravenous injection. The relatively clean spectrum of the control serum (Figure 3(a)) around the region of the molecular anion peak (550.3* m/z*) permitted us to follow the clearance of IG20 in adult rats (Figure 3). The IG20 signal was progressively decreasing but it was still detected at 24 h (Figures 3(a)–3(e)). The average of signal intensity values for IG20 peak in serum analysis of each group of rats resume the clearance of IG20 in rat serum. Then pharmacokinetic parameters such as half-life could be inferred using linear regression [11] in graph of Figure 3(f). Thus, we obtained that intact IG20 has a half-life of 9 h in serum of intravenous injected rats. Nevertheless, this information should be considered in an orientative manner since the analytical method was not optimized for quantitative measurements.

### 3.4. Monitoring of IG20 in CNS Tissue Homogenates by Mass Spectrometry

**Figure 4:**
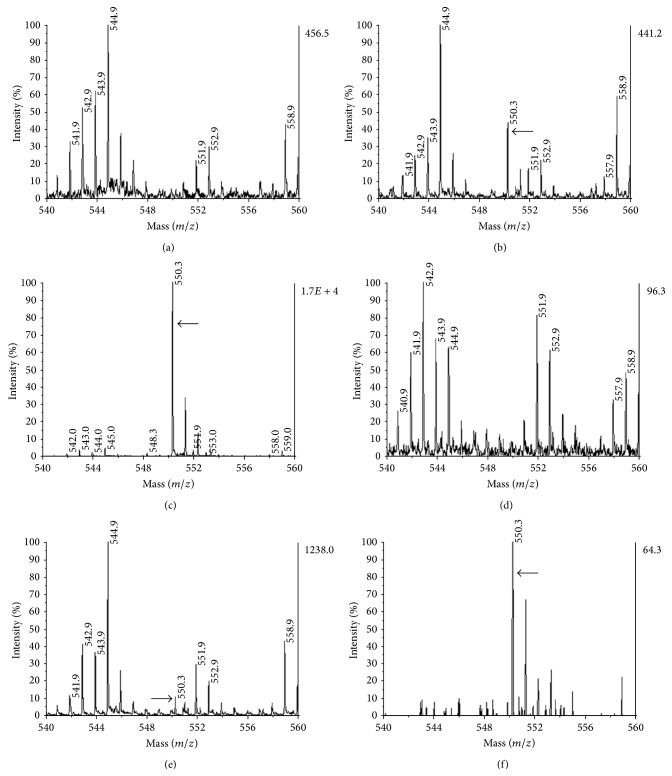
The IG20 is recovered from CNS tissues after deproteinization and lipid extraction. The mass spectrum of tissue homogenates and glycolipid extracts from spinal cord ((a); (b), (c)) or brain ((d); (e), (f)) is shown. In IG20 doped homogenates of spinal cord (a) or brain (d) no signal was obtained. The extraction of total glycolipids allowed detection of synthetic glycoside in spinal (b) and brain extracts (e). When tissue suspension was deproteinized by proteinase K prior to glycolipids extraction, a higher signal for IG20 compound was obtained ((c), (f)). Arrows correspond to mass-charge peak for IG20.

We also used mass spectrometry for detection of IG20 in brain and spinal cord tissues of treated animals (Figure 4). Initially, the spectra analysis of homogenates of CNS tissues from animals treated with IG20 did not show signal for this compound, neither in homogenized spinal cord or brain tissues that were doped with IG20 (Figures 4(a) and 4(d)). This led us to make changes in the processing conditions of the tissues samples and we continued using doped samples to circumvent a possible metabolic modification of IG20.

These doped samples when submitted to total glycolipids extraction (see Section 2) allowed us to detect glycoside in spinal cord and brain tissues, respectively (Figures 4(b) and 4(e)), though in lower amounts than expected. This result suggested that a significant amount of compound could be associated with proteins present in the tissues precluding its detection by mass spectrometry. Consequently we optimize this recovery achieved for IG20 when doped tissue suspension was deproteinized with proteinase K prior to glycolipids extraction, yielding a clean and strong mass signal for IG20 in spinal and, much less intensive, in brain tissue (Figures 4(c) and 4(f), resp.). The extractive method was quite more efficient in homogenates of spinal cord in comparison to brain extract (Figures 4(c) and 4(f)).

### 3.5. Low Recovering of Locomotion in SCI Rats that Were Intravenous Injected with IG20

**Figure 5:**
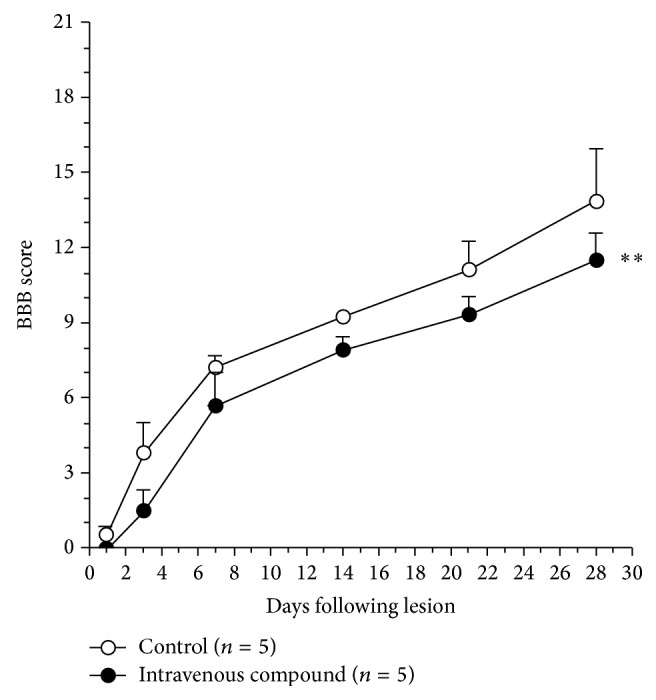
The intravenous administration of IG20 compound affects the recovery from SCI in locomotion rating scale for lesioned rats. The adult rats with a contusion in spinal cord were evaluated by BBB rating test in days postlesion as shown. The animals treated by IG20 and controls have shown the same deficit in locomotion at day 1, but the recovery was higher in the control group of saline than in treated animals during this study. ^∗∗^ *P* value = 0.0020.

The BBB test is broadly used in studies of recovery after spinal cord injury in rats [7, 12, 13]. We applied BBB scale in days after the lesion to rats with moderate contusion in spinal cord at thoracic level. At day 1 after spinal contusion the control and IG20 treated groups showed the same low recovery in BBB score. A continuous recovering in locomotion was observed in both groups; however, the recovery was more important in lesioned rats injected with saline than animals treated by IG20 (Figure 5). The low recovery in locomotion after SCI in IG20 group could be associated with the way used for IG20 administration, as is discussed.

The above results supported the use of mass spectrometry to monitoring sulfated compounds of glycolipid nature in serum, spinal cord, and brain with application to SCI preclinical studies.

## 4. Discussion and Conclusions 

The mass spectrometry (MS) has experienced great progress over the last years in the biomedicine field, and its use in spinal cord pathologies or therapies is increasing [14–17]. The MS has been reported in glycomic analysis of central nervous system [18–20] and different studies of glycosides related to spinal cord [21–23]. In this sense the use MS technology to study the effects of dexamethasone in patients with spinal cord injury (SCI) [24] and models of pain or multiple sclerosis in rats [25, 26] has been described. Determination of this glucocorticoid drug by MS after intravenous injection has been also described [27]. These works pointed that MS is a useful tool in SCI drug development, and particularly to detect and monitoring the new drugs in cells and tissues from CNS.

**Figure 6:**
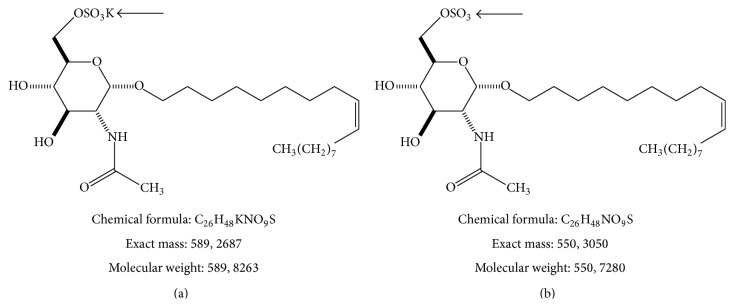
The structure of IG20 glycoside (a) and its sulfuric form (b). The change in elemental composition, exact mass and reduction in molecular weight by mass detection in negative-ion mode is shown. Arrows pointed the presence/absence of potassium in the IG20 structure.

After SCI is produced a glial scar is generated in the medullar zone affected. This forms a barrier for growing axons, myelination, and recovery from SCI. The astroglia and microglia are principal cell components of glial scar, and the use of molecules able to inhibit their proliferation represents an approach for SCI treatment [28–30]. We have described the chemical synthesis and antiglioma activity of synthetic glycosides [6, 31]. Glycoside IG20 (Figure 6) has showed chemical stability, solubility in polar solvents and high inhibitory capacity over glioma growth. Now we observed that IG20 was capable of inhibiting the astroglia and microglia cells in the low micromolar range (Table 1). This glial inhibition could be produced by interaction with the product of Arhgdia gene (RhoGDI*α*), a RhoGTPase regulator, like was observed for neurostatin and NF115 analogues in human glioma cells [31].

In the present study we choose matrix-assisted laser desorption/ionization (MALDI) as ionization method and time of flight (TOF) as detection for MS in order to monitor the IG20 in animal samples. MALDI has several favorable attributes like very high levels of sensitivity, high throughput by using sample plates that are loaded with more than 100 different samples, and high tolerance to salts and buffers [32, 33]. Nowadays, there is an increasing interest in performing systemic analyses and MALDI continues to be a major technique for the analysis of carbohydrates [34]. During our screening for IG20 content a large number of samples must be analyzed, that is why fast and reliable analytical methods were required. MALDI-MS is an attractive alternative to ESI-MS since it is more tolerant towards salts and therefore does not require extensive desalting before analyzing the sample. The MALDI-TOF MS methods are becoming quicker in screening tasks, and more recently it is being used for quantitative analysis of polysaccharides [35–37].

In the MALDI mass spectrum we obtained a high intensity signal and distinctive mass for IG20 (550.3* m/z*) in ion-negative mode. This made a good discrimination of our compound from ionized components of matrix. In a more restricted range of mass-to-charge (545 to 555) the IG20 isotopic distribution and the deisotoping permitted us to verify the presence of compound in the serum and tissues samples. The rapid and simple processing of serum samples for MS analysis makes this procedure attractive for synthetic glycoside detection and we recommend its use for pharmacokinetic studies as an alternative to MS monitoring in plasma samples [38]; but a good discrimination from background must be achieved previously [39].

Mass spectrometry, in combination with separation techniques, can play an important role in identifying and monitoring biomarkers in physiological fluids, which is a useful way of assessing drug efficacy and safety issues [32]. For example, pharmacokinetic studies where tandem of chromatographic purification/MS was applied to monitoring the molecule of interest have been carried out [38, 40–42]. Here we detected the IG20 in samples of serum and CNS tissues from animals injected with IG20. In serum samples the time of clearance (24 h) and half-life of intact compound (4 h) are pharmacokinetic indicatives that IG20 compound is available in blood circulation without modification by cellular or enzymatic components of blood. This offers a window of time that must be useful in future preclinical trials, previous to SCI therapeutic treatment.

The principal source of lipids in nervous system is myelin and the lipidic components of neural cell membranes. The lipid composition in CNS is similar in spinal cord and brain where oligodendrocytes are the myelin producing cells; but there is some difference with Peripheral Nervous System where Schwann cells produce the peripheral myelin [37]. In consonance to this, we obtained similar mass spectra in the initial lipid extracts from brain and spinal cord, although the size of peaks was not the same (Figures 4(a) and 4(d)). A similar proportion in the lipid content between brain and spinal cord could mean that IG20 glycolipid distribution is also similar for both CNS tissues on* in vivo* conditions.

On the other hand, in animals treated with intravenous injection of IG20 no peak corresponding to IG20 was detected in mass spectra of homogenates of CNS tissue samples. In order to circumvent the brain blood barrier we included IG20 doped samples as control. The absence of IG20 signal in doped samples suggested that IG20 could be complexes with some components of CNS tissues; see Figure 4. The signal for IG20 was however observed after deproteinization and lipid extraction of brain and spinal cord doped samples. We can speculate that IG20 is interacting with a protein(s), like RhoGDI*α* in glial cells [31], and this protein(s) could be the target for this compound in brain and spinal cord.

Our final objective is to find a drug(s) for SCI treatment. For this purpose we used a moderate spinal contusion model and the BBB test to registering the motor recovery after spinal lesion in rats [7]. We observed that animals intravenously injected with IG20 fail to recover from spinal lesion, in comparison to control of saline injected group; see Figure 5. The systemic way of drug administration and blood brain barrier could be contribute to this poor motor recovery in IG20 treated animals. We suggested that local administration of IG20 could be circumvent this low locomotion recovery after SCI. The direct inhibition by IG20 of resident astroglia and microglia cells, principal components of glia scar, may be obtained if the drug is administered close to the zone of spinal lesion.

In conclusion, in this work we have used mass spectrometry to monitor the IG20 synthetic compound in cells, serum and tissues of CNS, as part of preclinical studies for spinal cord injury treatment. The pharmacokinetic parameters, clearance at 24 h, and half-life at 9 h, for this compound in adult rat, were achieved. The deproteinization and lipid extraction allowed the detection of IG20 by mass spectrometry. In our experience, the mass spectrometry is a powerful tool to the research and development of drugs to treat pathologies of CNS.

## Supplementary Material

Supplementary Material contains 3 Figures that shows some relevant information to this work. In Figure 1, the circles represent the ways used to pick out samples in the plate during MALDI-TOF MS technique, and its respective mass spectrums (arrow heads for IG20 signal). In Figure 2, the qualitative compound report for IG20 as final product, and the ESI-MS analysis. Finally in Figure 3, the gaining weight evolution in rats injected with IG20 every two days; the concentration of 30 mg/kg was selected for experiments.

## Figures and Tables

**Table 1:** The IC_50_ inhibition values of microglia and astroglia by IG20 glycosides.

Cells	Origin	Glycoside	IC_50_ (*µ*M)^*^
BV-2	Mouse microglia	IG20	21 ± 3.7
N13	Mouse microglia	IG20	5.7 ± 0.1
Astrocyte	Rat astroglia	IG20	9.3 ± 0.7

^∗^IC_50_  ± SE.

## Abbreviations

CNS: Central nervous system
SCI: Spinal cord injury
MALDI: Matrix-assisted laser desorption/ionization
ESI: Electrospray ionization.
