# Supplementary material for: Mass Spectrometry in Pharmacokinetic Studies of a Synthetic Compound for Spinal Cord Injury Treatment
Source: Biomed Res Int. 2015 May 19;2015:169234. doi: 10.1155/2015/169234 (PMC4452236; doi:10.1155/2015/169234)
Supplement: Supplementary file 1 — Supplementary Material contains 3 Figures that shows some relevant information to this work. In Figure 1, the circles represent the ways used to pick out samples in the plate during MALDI-TOF MS technique, and its respective mass spectrums (arrow heads for IG20 signal). In Figure 2, the qualitative compound report for IG20 as final product, and the ESI-MS analysis. Finally in Figure 3, the gaining weight evolution in rats injected with IG20 every two days; the concentration of 30 mg/kg was selected for experiments. [file 169234.f1.pdf]

Supporting information for

Mass spectrometry in pharmacokinetic studies of a synthetic compound for spinal cord injury treatment.

María Sánchez-Sierra[1], Isabel García-Álvarez[1], Alfonso Fernández-Mayoralas[2], Sandra Moreno-Lillo[1], Gemma Barroso García[3], Verónica Moral Dardé[3], Ernesto Doncel-Pérez [1*]

1) Grupo de Química Neuro-regenerativa, Hospital Nacional de Parapléjicos, Servicio de Salud de Castilla La Mancha (SESCAM), Finca La Peraleda s/n, 45071 Toledo, Spain.

2) Instituto de Química Orgánica General, CSIC, Juan de la Cierva 3, 28006 Madrid, Spain.

3) Servicio de Proteómica, Hospital Nacional de Parapléjicos, Servicio de Salud de Castilla La Mancha (SESCAM), Finca La Peraleda s/n, 45071 Toledo, Spain.

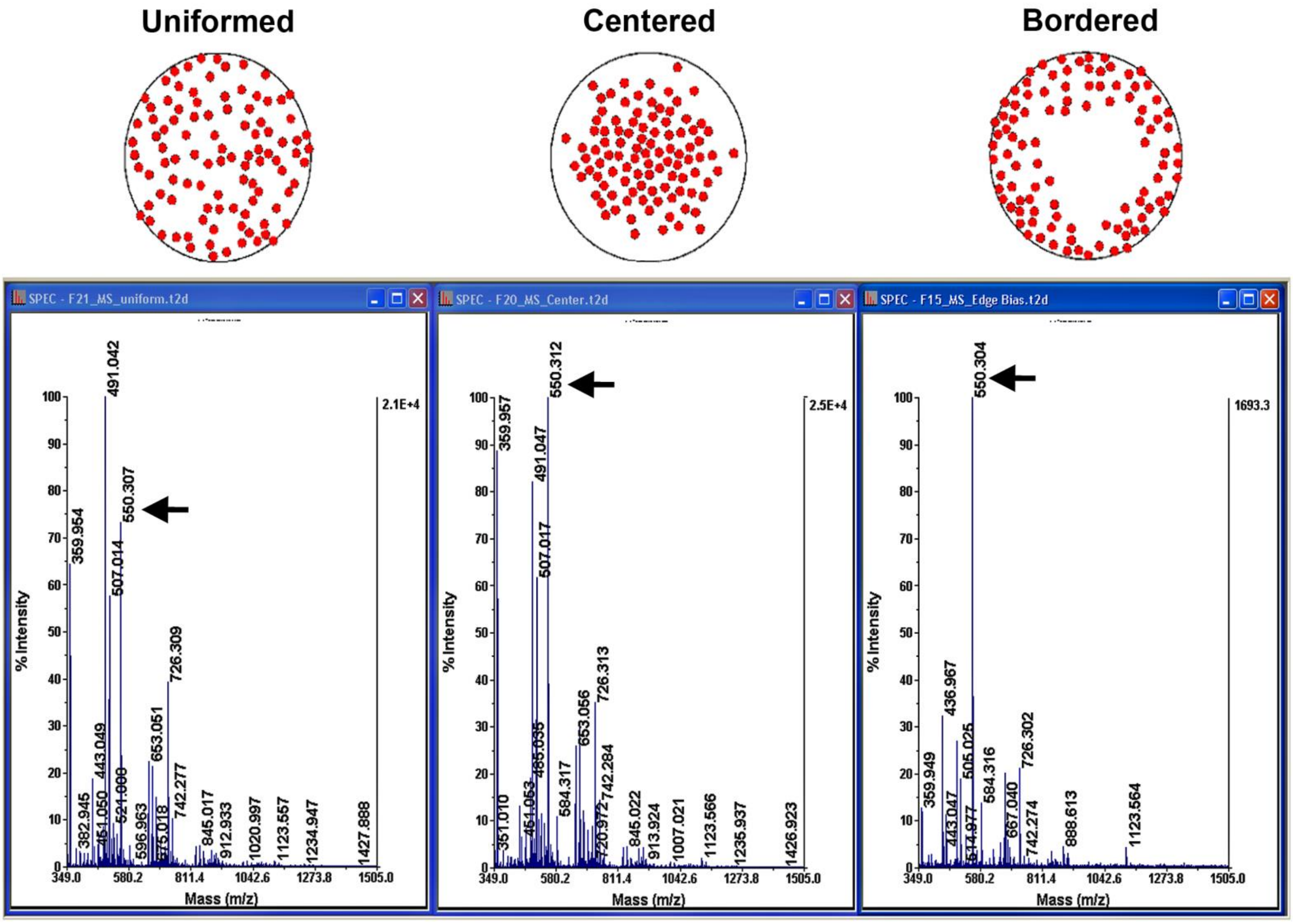

Figure 1 Examples of acquisition of samples in MALDI-TOF MS analysis in this study. In circles ways of sample acquisition, below examples of mass spectrums in each case. Arrows for IG20 signal at 550,3 m/z.

Qualitative Compound Report

**Data File**	6287_ig_20_oct_2013_01.d	**Sample Name**	Unavailable
**Sample Type**	Unavailable	**Position**	Unavailable
**Instrument Name**	Unavailable	**User Name**	Unavailable
**Acq Method**		**IRM Calibration Status**	Success
**DA Method**	QualDAMethod.m	**Comment**	Sample information is unavailable

Compound Table

Compound Label	RT	Mass	Abund	Formula	Tgt Mass	Diff (ppm)
Cpd 1: C26 H48 N O9 S	0.612	550.3039	17028	C26 H48 N O9 S	550.305	-1.92

Compound Label	RT	Algorithm	Mass
Cpd 1: C26 H48 N O9 S	0.612	Find By Formula	550.3039

MS Zoomed Spectrum

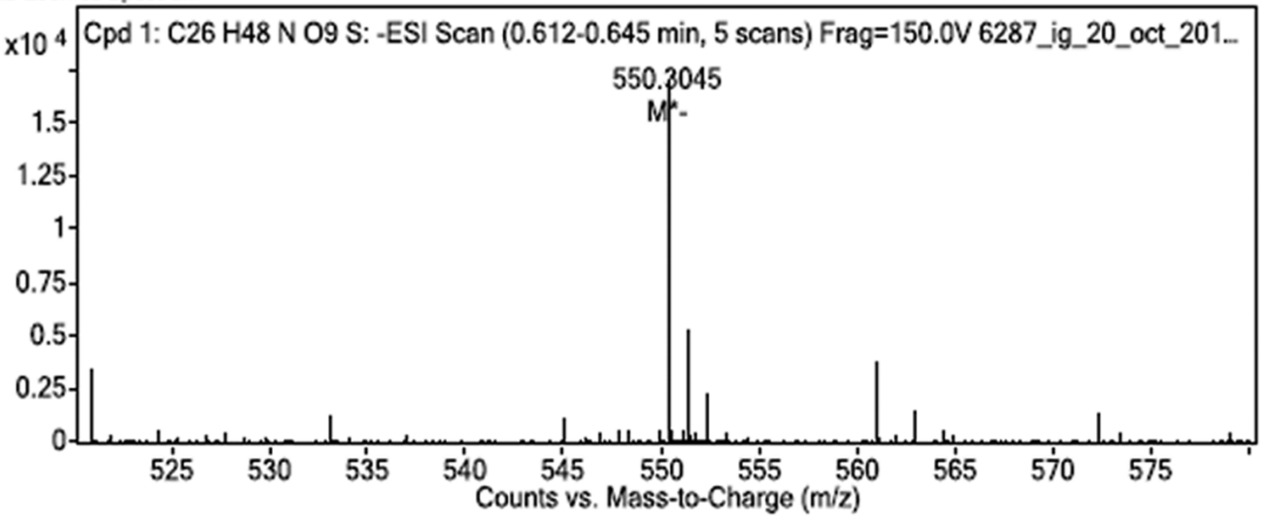

MS Spectrum Peak List

m/z	*Calc m/z*	Diff(ppm)	z	Abund	Formula	Ion
112.9855				1030501		
113.0529				26526		
113.0819				79022		
113.1143				13098		
113.1392				8905		
113.1597				6448		
113.1835				6726		
113.2082				7051		
113.988				35159		
550.3045	550.3055	-1.88	1	17028	C26 H48 N O9 S	M*-

--- End Of Report ---

Figure 2. Qualitative compound report for IG20 by ESI-MS analysis. The final product in the IG20 synthesis was analyzed by Electrospray Ionization Mass Spectrometry, in ion negative mode. The mass/charge at 550.30 and purity of IG20 is shown.

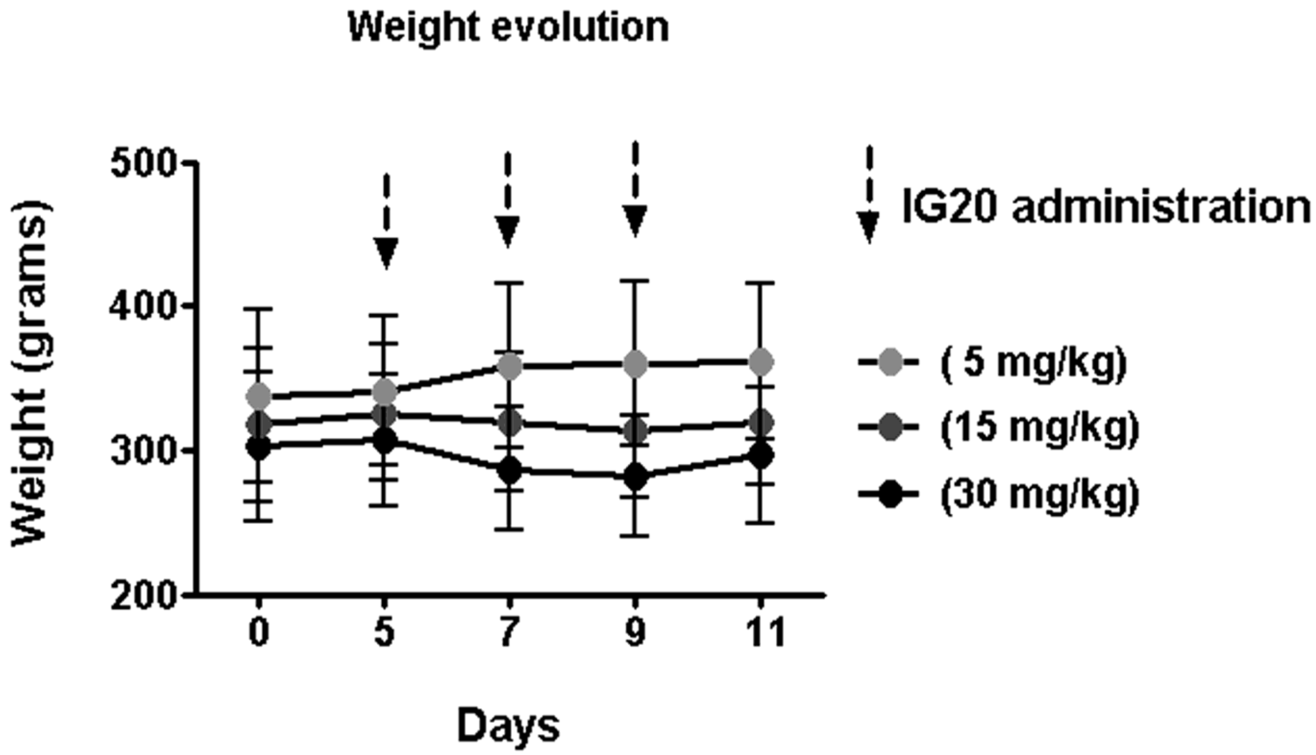

Figure 3. Evolution of weight in rats injected with IG20 compound. Normal adult rats were injected every two days with different concentrations of IG20 compound. The gaining of weight was used as a health parameter. There was not significant difference in between groups of animals during the period assayed. The higher concentration of IG20 (30mg/kg) was selected for successive experiments.
